# Plant growth-promotion and biofortification of chickpea and pigeonpea through inoculation of biocontrol potential bacteria, isolated from organic soils

**DOI:** 10.1186/s40064-016-3590-6

**Published:** 2016-10-26

**Authors:** Subramaniam Gopalakrishnan, Srinivas Vadlamudi, Srinivasan Samineni, C. V. Sameer Kumar

**Affiliations:** International Crops Research Institute for the Semi-Arid Tropics (ICRISAT), Patancheru, Telangana 502 324 India

**Keywords:** Plant growth-promotion, Biofortification, Bacteria, Chickpea, Pigeonpea

## Abstract

Seven strains of bacteria [*Pseudomonas plecoglossicida* SRI-156, *Brevibacterium antiquum* SRI-158, *Bacillus altitudinis* SRI-178, *Enterobacter ludwigii* SRI-211, *E. ludwigii* SRI-229, *Acinetobacter tandoii* SRI-305 and *Pseudomonas monteilii* SRI-360; demonstrated previously for control of charcoal rot disease in sorghum and plant growth-promotion (PGP) in rice] were evaluated for their PGP and biofortification traits in chickpea and pigeonpea under field conditions. When treated on seed, the seven selected bacteria significantly enhanced the shoot height and root length of both chickpea and pigeonpea over the un-inoculated control. Under field conditions, in both chickpea and pigeonpea, the plots inoculated with test bacteria enhanced the nodule number, nodule weight, root and shoot weights, pod number, pod weight, leaf weight, leaf area and grain yield over the un-inoculated control plots. Among the seven bacteria, SRI-229 was found to significantly and consistently enhance all the studied PGP and yield traits including nodule number (24 and 36%), nodule weight (11 and 44%), shoot weight (22 and 20%), root weight (23 and 16%) and grain yield (19 and 26%) for both chickpea and pigeonpea, respectively. When the harvested grains were evaluated for their mineral contents, iron (up to 18 and 12%), zinc (up to 23 and 5%), copper (up to 19 and 8%), manganese (up to 2 and 39%) and calcium (up to 22 and 11%) contents in chickpea and pigeonpea, respectively, were found enhanced in test bacteria inoculated plots over the un-inoculated control plots. This study further confirms that the selected bacterial isolates not only have the potential for PGP in cereals and legumes but also have the potential for biofortification of mineral nutrients.

## Background

The United Nations (UN) announced 2016 as ‘International Year of Pulses (2016 IYOP)’ in order to emphasize the need to focus on pulses as critical components for global food and nutritional security and to create awareness and understanding of the challenges faced in pulse farming. Pulses, are often referred to as “poor mans’ meat”, as they offer a cost-effective alternative to animal proteins. Besides the protein, their richness in micronutrients and other vital elements including amino acids make pulses as critical entities in food and feed value chains around the world. Chickpea (*Cicer arietinum* L.) and pigeonpea (*Cajanus cajan* [L.] Millsp.) are the two most important pulse crops grown under semi-arid tropical conditions and their production is hindered by biotic and abiotic constraints, including insect and disease incidence, infertile soils and climate variability. Global yields of both chickpea (968 kg ha^−1^) and pigeonpea (762 kg ha^−1^) have been relatively stagnant (FAOSTAT 2013) for the last five decades in spite of using various conventional and molecular breeding approaches and extensive use of inorganic fertilizers and pesticides that created environmental and health concerns. With the ever increasing cost of pesticides and fertilizers and concern over environmental degradation, there has been a resurgence of interest to develop environment-friendly methods of crop production and protection (Jannouraa et al. 2013). The environment-friendly options include the use of plant growth-promoting (PGP) microbes, antagonistic or entomopathogenic microbes, animal wastes, botanicals and crop residues serves as an alternative to chemical fertilizers and pesticides (Rupela et al. 2005).

PGP bacteria are well known for their usefulness in crop production and protection and in maintaining soil health. They are commonly found in soil, compost, fresh and marine water and decomposing organic materials and produce secondary metabolites with agricultural importance. PGP bacteria has been cited not only to improve plant growth but also to suppress the insect pests and plant pathogens, of which *Bacillus* spp., *Pseudomonas* spp., *Enterobacter* spp., *Brevibacterium* spp. and *Streptomyces* spp. are important (Weller et al. 2002; Singh et al. 2008; Soe et al. 2010; Sreevidya and Gopalakrishnan 2016). PGP bacteria are also reported to enhance micronutrient uptake (mainly on iron and zinc) in the harvested grains of various crops including rice and wheat (Rana et al. 2012a, b; Sharma et al. 2013). This is achieved through the synthesis of various molecules such as siderophores, organic acids and exopolysaccharides by the PGP bacteria for increasing the mineral availability in the root-soil interface and further mobilization into plants. Exploration of such potential PGP bacteria offer the prospect of alternative chemical crop protection agents and improved environmental health and sustainability and thereby gives an option to use extensively in organic agriculture.

Seven bacteria, (*Pseudomonas plecoglossicida* SRI-156, *Brevibacterium antiquum* SRI-158, *Bacillus altitudinis* SRI-178, *Enterobacter ludwigii* SRI-211, *E. ludwigii* SRI-229, *Acinetobacter tandoii* SRI-305 and *P. monteilii* SRI-360), isolated from the rhizospheres of an organically grown system of rice intensification (SRI) fields, were earlier reported by us for biocontrol of charcoal rot of sorghum, caused by *Macrophomina phaseolina* (Tassi) Goid. and PGP in sorghum and rice (Gopalakrishnan et al. 2011, 2012). The major objectives of the present study were to further evaluate the PGP and biofortification potentials of the seven bacteria on grain legumes such as chickpea and pigeonpea.

## Methods

### Bacterial strains

Seven bacteria isolated from rhizosphere of SRI organic fields, SRI-156 (*P. plecoglossicida*; NCBI accession: JQ247008), SRI-158 (*B. antiquum*; NCBI accession: JQ247009), SRI-178 (*B. altitudinis*; NCBI accession: JQ247010), SRI-211 (*E. ludwigii*; NCBI accession: JQ247011), SRI-229 *(E. ludwigii*; NCBI accession: JQ247012), SRI-305 (*A. tandoii*; NCBI accession: JQ247013) and SRI-360 (*P. monteilii*; NCBI accession: JQ247014), reported previously as potential for biocontrol and PGP traits in sorghum and rice by us (Gopalakrishnan et al. 2011, 2012), were further investigated in this study.

### Influence of selected bacterial strains on seed germination and seedling growth of chickpea and pigeonpea

Seeds of chickpea (variety ICCV 2; matures at 85–90 days and yields 1.1–1.2 t ha^−1^) and pigeonpea (variety ICPL 88039; matures at 120 days and yields about 1.5 t ha^−1^) were surface sterilized (with 2% sodium hypochlorite solution for 5 min and rinsed with sterilized water for five times). One hundred numbers of surface-sterilized seeds of chickpea and pigeonpea were soaked separately with the seven selected bacteria (grown separately in nutrient broth, NB) for 40 min (10^8^ CFU ml^−1^). At the end of soaking, the seeds were sown on disposable tea cups containing un-sterilized sand. The whole set up was kept at 26 °C in a greenhouse for 10 days. Water was added if required. At the end of the incubation, % germination, shoot heights and root lengths were noted.

### Influence of selected bacterial strains for PGP potential on chickpea and pigeonpea under field conditions

The field trials of chickpea was carried out in 2013–2014 at ICRISAT, Patancheru (17°30′N; 78°16′E; altitude 549 m), in the Telangana state of India. Soils at the field site are classified as Vertisols (containing 51% clay, 27.5% sand and 21.5% silt) with an alkaline pH (7.7–8.3) and an OC content of 0.5–0.6%. The mineral content of the top 15 cm rhizosphere soil include, 24 mg kg^−1^ soil of available N, 9 mg kg^−1^ soil of available P and 290 mg kg^−1^ soil of available K. At 3 days before sowing, 18 kg N ha^−1^ and 20 kg P ha^−1^ as di-ammonium phosphate (DAP) were incorporated in the soil. The trial was laid out in a RCBD design with three replicates and subplot sizes of 4 m × 3 ridges. The seven selected bacterial strains (SRI-156, SRI-158, SRI-178, SRI-211, SRI-229, SRI-305 and SRI-360) were cultured individually on NB at 28 °C for 48 h. The seeds of chickpea (ICCV 2) were treated with the bacterial strains (individually; containing 10^8^ CFU ml^−1^) for 40 min and sown immediately by hand planting on 2 November 2013 in rows 30 cm apart at a depth of 5 cm to achieve an estimated plant population of at least 26 plants m^−2^. Plants were inoculated with respective bacterial strains until the flowering stage once every 15 days on the soil close to the plant. Control plots were not treated with bacteria. No pesticide was sprayed during the cropping period, as no serious phytopathogens or insect pest attacks were observed. The crop was manually harvested on 10 Feb 2014. At 30 days after sowing (DAS), the nodule number, nodule weight, root weight and shoot weight were noted while at 60 DAS, the pod number, pod weight, leaf area and leaf weight were noted. At crop maturity, the stover yield, grain yield, pod weight, pod number and seed weight were noted.

The field trial of pigeonpea was carried out in the rainy season of 2014 at ICRISAT, Patancheru on Vertisols. The experiment was laid out with three replicates and subplot sizes of 4 m × 2 ridges (1.2 m) in a RCBD design. Seeds of pigeonpea (variety ICPL 88039) were treated individually with the selected bacterial strain (10^8^ CFU ml^−1^) for 45 min and sown by dibbling (in rows 60 cm apart and 10 cm between plants at a depth of 5 cm) on 28 June 2014. Plants were inoculated with bacterial strains until the flowering stage once every 15 days on the soil close to the plant. Control plots were not treated with bacterial strains. All the agronomic practices including weeding and irrigation were done as and when required. The crop was harvested manually on 27 Oct 2014. At 30 DAS, the nodule number and nodule weight while at 60 DAS, the branches number, leaf area, leaf weight, stem weight and root weight were recorded. At crop maturity, the plant height, shoot weight, pod weight, pod number, seed weight, seed number, stover yield and grain yield were recorded.

At crop maturity, rhizosphere soil samples from both chickpea and pigeonpea were collected from the top 15 cm depth of the soil profile and analyzed for soil nutrients such as total nitrogen, available phosphorous and organic carbon as per the protocols of Novozamsky et al. (1983), Olsen and Sommers (1982) and Nelson and Sommers (1982), respectively.

### Influence of selected bacterial strains on micronutrients concentration in harvested grains of chickpea and pigeonpea

Both chickpea and pigeonpea harvested grains were dried and ground at room temperature (30 ± 2 °C) and digested using nitric acid and hydrogen peroxide system as per AOAC (2000). The digested samples were analyzed for micronutrients concentration using inductively coupled plasma-optical emission spectroscopy (ICP-OES) by the Prodigy High Dispersion ICP-OES instrument (Teledyne Leeman Labs) against known standards. For testing the availability of micronutrients in the edible form i.e. cooked grains, the harvested grains were soaked in water at 1:10 ratio of grains: water (w:v) for 8 h and subjected to autoclaving at 121 °C for 15 min. The autoclaved seeds were drained from excess water and allowed to dry at 30 ± 2 °C. The processed dried grains were ground into fine powder, digested and analysed for micronutrient analysis as described earlier.

### Statistical analysis

Data were analysed by analysis of variance (ANOVA) and the GLM (General Linear Model) procedure in the software package SAS (SAS Inst. 2002–2008, SAS V9.3), considering isolates and replication as fixed in RCBD. Isolate means were tested for significance and compared using Fisher’s protected least significant difference (LSD) test.

## Results

### Influence of selected bacterial strains on seed germination and seedling growth

**Fig. 1:**
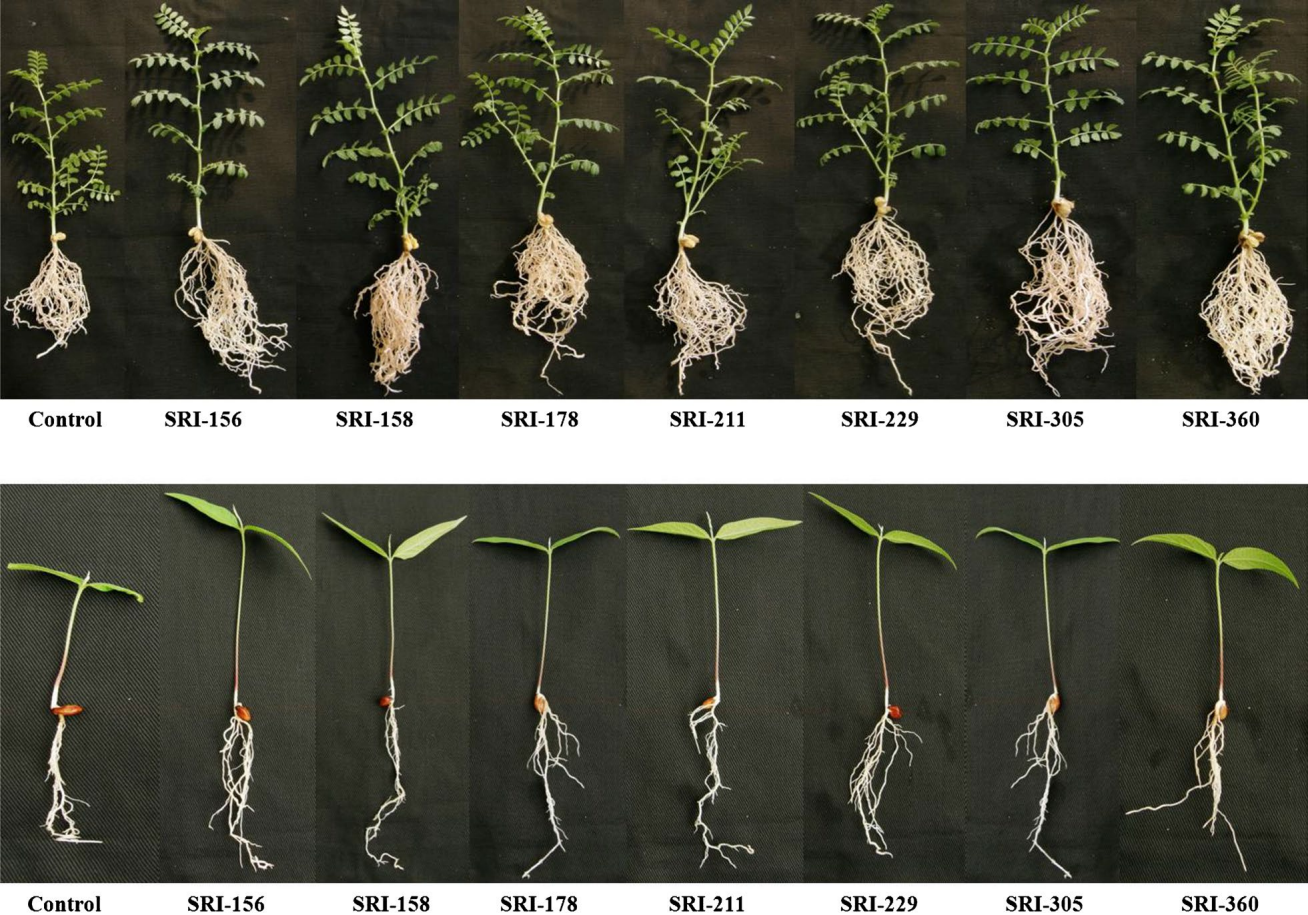
Influence of selected bacterial strains on germination of chickpea and pigeonpea seed

The selected seven bacterial strains did not show any influence on germination as 100% germination was found in both bacteria-treated as well as untreated control plants in both chickpea as well as pigeonpea. However, the shoot height and root lengths of seedlings were increased significantly up to 17 and 30%, respectively for chickpea and up to 29 and 22%, respectively for pigeonpea (Table 1; Fig. 1). Among the bacterial treatments, SRI-229 and SRI-305 in chickpea and SRI-158 and SRI-211 in pigeonpea were found to increase both shoot height and root length significantly (*p* < 0.05) over the un-inoculated control. Other bacterial treatment showed either increased shoot height or root length. As a whole, the bacterial treatments significantly increased the shoot height rather than root length in both the legumes over the control.

**Table 1:** Influence of selected bacterial strains on germination of chickpea and pigeonpea seed

Bacteria	Chickpea		Pigeonpea	
	Shoot height (cm)	Root length (cm plant^−1^)	Shoot height (cm)	Root length (cm plant^−1^)
SRI-156	18.9*	14.4^NS^	13.3*	10.3^NS^
SRI-158	17.3^NS^	14.9^NS^	14.8*	12.5*
SRI-178	19.0*	14.8^NS^	13.2*	10.4^NS^
SRI-211	18.8*	15.0^NS^	13.4*	12.7*
SRI-229	19.2*	19.5*	12.8*	11.1^NS^
SRI-305	17.6*	19.9*	12.3^NS^	9.9^NS^
SRI-360	17.7*	18.0^NS^	12.2^NS^	9.9^NS^
Control	15.9	13.9	10.5	9.8
Mean	18.0	16.3	12.8	10.8
SE±	0.50	1.43	0.70	0.61
LSD (5%)	1.52	4.33	2.16	1.89
CV %	5	15	10	10

*SE* standard error, *CV* coefficients of variation, *NS* non-significant

* Indicates significance over the control at *p* < 0.05

### Influence of selected bacterial strains for PGP potentials under field conditions

The chickpea plots treated with the seven selected bacterial strains enhanced agronomic performance of all the PGP traits measured including the nodule number (up to 24%), nodule weight (up to 14%), root weight (up to 25%) and shoot weight (up to 22%) at 30 DAS and the pod number (up to 39%), pod weight (up to 30%), leaf weight (up to 24%) and leaf area (up to 24%) at 60 DAS over the un-inoculated control plots (Table 2). Among the bacterial treatments, SRI-229 was found to increase all the measured parameters significantly (*p* < 0.05) at 30 and 60 DAS, followed by SRI-158. As a whole, pod weight followed by nodule weight was increased significantly (*p* < 0.05) by the bacterial treatments. At crop maturity, the chickpea plots treated with the bacterial strains enhanced yield traits including the stover yield (up to 25%), grain yield (up to 19%), pod weight (up to 39%), pod number (up to 33%) and seed weight (up to 29%). Among the bacterial treatments, SRI-158, SRI-229 and SRI-305 were found to significantly (*p* < 0.05) increase the yield traits including stover yield, grain yield, pod weight, pod number and seed weight over the un-inoculated control. SRI-360 alone didn’t produce any significant increase on yield traits Table 3). The soil nutrients including total N (up to 19%), available P (up to 27%) and OC % (up to 8%) were found to be increased over the un-inoculated control; however, the significance was noticed only on three bacterial treatments (SRI-178, SRI-211 and SRI-229) for OC, two bacterial treatments (SRI-178 and SRI-305) for available P and one bacterial treatment (SRI-158) for total N (Table 4).

**Table 2:** Effect of the seven biocontrol potential bacteria on the agronomic performance of chickpea under field conditions—at 30 and 60 days after sowing

Isolate	30 days after sowing				60 days after sowing			
	Nodule number (plant^−1^)	Nodule weight (mg plant^−1^)	Root weight (mg plant^−1^)	Shoot weight (g plant^−1^)	Pod number (plant^−1^)	Pod weight (g plant^−1^)	Leaf weight (g plant^−1^)	Leaf area (m^−2^)
SRI-156	49^NS^	225^NS^	177^NS^	1.74^NS^	74^NS^	5.09*	4.38*	709^NS^
SRI-158	56^NS^	257*	223*	1.89^NS^	70^NS^	5.99*	4.81*	792*
SRI-178	53^NS^	230^NS^	179^NS^	1.75^NS^	97*	5.73*	4.46*	828*
SRI-211	50^NS^	237*	169^NS^	1.87^NS^	70^NS^	5.48*	3.72^NS^	657^NS^
SRI-229	65*	248*	219*	2.21*	79*	5.02*	4.85*	765*
SRI-305	59*	238*	173^NS^	2.09*	74^NS^	5.94*	4.00^NS^	713^NS^
SRI-360	55^NS^	239*	176^NS^	1.76^NS^	66^NS^	4.60^NS^	3.55^NS^	643^NS^
Control	49	221	168	1.72	59	4.21	3.71	632
Mean	55	237	186	1.88	74	5.26	4.19	717
SE±	2.5	4.9	7.9	0.086	6.2	0.206	0.166	41.2
LSD (5%)	7.6	14.8	24.1	0.261	18.8	0.624	0.503	125.1
CV %	8	4	7	8	15	7	7	10

*SE* standard error, *CV* coefficients of variation, *NS* non-significant

* Indicates significance over the control at *p* < 0.05

**Table 3:** Effect of the seven biocontrol potential bacteria on yield performance of chickpea under field conditions—at harvest

Isolate	Stover yield (t ha^−1^)	Grain yield (t ha^−1^)	Pod weight (g plant^−1^)	Pod number (plant^−1^)	Seed weight (g plant^−1^)
SRI-156	1.70^NS^	1.79*	21.4*	79*	15.85*
SRI-158	2.19*	1.79*	28.1*	89*	17.54*
SRI-178	1.73^NS^	1.92*	18.8^NS^	68^NS^	14.08^NS^
SRI-211	1.65^NS^	1.78*	18.3^NS^	70^NS^	13.95^NS^
SRI-229	1.82*	2.02*	22.4*	80*	16.41*
SRI-305	1.91*	1.86*	24.6*	98*	18.61*
SRI-360	1.65^NS^	1.71^NS^	17.2^NS^	66^NS^	13.30^NS^
Control	1.65	1.63	17.0	65	13.30
Mean	1.79	1.81	21.0	77	15.38
SE±	0.055	0.048	1.00	2.3	0.592
LSD (5%)	0.168	0.145	3.04	7.1	1.795
CV %	5	5	8	5	7

*SE* standard error, *CV* coefficients of variation, *NS* non-significant

* Indicates significance over the control at *p* < 0.05

**Table 4:** Effect of the seven biocontrol potential bacteria on rhizosphere soil nutrients of chickpea under field conditions—at harvest

Isolate	Total N (ppm)	Available P (ppm)	Organic carbon (%)
SRI-156	739^NS^	6.85^NS^	0.55^NS^
SRI-158	872*	6.65^NS^	0.57^NS^
SRI-178	741^NS^	9.00*	0.58*
SRI-211	748^NS^	6.55^NS^	0.60*
SRI-229	737^NS^	7.10^NS^	0.58*
SRI-305	717^NS^	8.20*	0.56^NS^
SRI-360	735^NS^	6.75^NS^	0.55^NS^
Control	710	6.55	0.55
Mean	750	7.21	0.56
SE±	13.6	0.390	0.009
LSD (5%)	45.6	1.309	0.029
CV %	3	8	2

*SE* standard error, *CV* coefficients of variation, *NS* non-significant

* Indicates significance over the control at *p* < 0.05

The pigeonpea plots treated with the seven bacterial strains also enhanced all the PGP traits measured including the nodule number (up to 38%) and nodule weight (up to 44%) at 30 DAS and number of branches (up to 25%), leaf weight (up to 26%), leaf area (up to 42%), stem weight (up to 27%) and root weight (up to 29%) at 60 DAS over the un-inoculated control plots (Table 5). As a whole nodule number was increased significantly (*p* < 0.05) by the bacterial treatments. Among the bacterial treatments SRI-229 was found to be the best followed by SRI-178. At crop maturity, the pigeonpea plots treated with the bacterial strains enhanced yield traits including the pod weight (up to 12%), pod number (up to 17%), seed weight (up to 21%), seed number (up to 23%), grain yield (up to 29%) and stover yield (up to 32%); still significant increases of grain yield was noticed only on SRI-158, SRI-178 and SRI-229 treatments over the un-inoculated control (Table 6). The soil nutrients including total N (up to 11%), available P (up to 38%) and OC % (up to 17%) were also found to be increased over the un-inoculated control (Table 7). Among the soil nutrients, OC % was found to be significantly (*p* < 0.05) increased by all the bacterial treatments except SRI-178 followed by available P and total N over the un-inoculated control. Among the bacterial treatments, SRI-360 was found to significantly (*p* < 0.05) increase all the three soil nutrient traits.

**Table 5:** Effect of the seven biocontrol potential bacteria on agronomic performance of pigeonpea under field conditions—at 30 and 60 days after sowing

Isolate	30 Days after sowing		60 Days after sowing				
	Nodule number (plant^−1^)	Nodule weight (g plant^−1^)	Branches number (plant^−1^)	Leaf weight (g plant^−1^)	Leaf area (m^−2^)	Stem weight (g plant^−1^)	Root weight (g plant^−1^)
SRI-156	5.1^NS^	1.3^NS^	10.3^NS^	8.96^NS^	1883^NS^	10.07^NS^	3.09^NS^
SRI-158	6.1*	2.0^NS^	11.7*	11.06*	2424*	11.45*	3.54^NS^
SRI-178	6.7*	2.0^NS^	9.3^NS^	9.08^NS^	1821^NS^	9.49^NS^	3.15^NS^
SRI-211	5.7*	1.7^NS^	10.3^NS^	8.92^NS^	2583*	10.34^NS^	3.14^NS^
SRI-229	6.5*	2.3*	10.7*	10.76*	2108*	11.43*	3.53^NS^
SRI-305	4.2^NS^	1.3^NS^	12.0*	11.77*	2711*	12.38*	4.18*
SRI-360	6.5*	1.8^NS^	11.0*	8.77^NS^	1731^NS^	9.17^NS^	3.35^NS^
Control	4.1	1.3	9.0	8.73	1576	9.10	2.98
Mean	5.6	1.7	10.5	9.76	2105	10.43	3.47
SE±	0.44	0.26	0.48	0.64	146.4	0.66	0.19
LSD (5%)	1.33	0.78	1.46	1.95	444.2	2.00	0.59
CV %	14	27	8	11	12	11	10

*SE* standard error, *CV* coefficients of variation, *NS* non-significant

* Indicates significance over the control at *p* < 0.05

**Table 6:** Effect of the seven biocontrol bacteria on yield performance of pigeonpea under field conditions—at harvest

Isolate	Pod weight (g plant^−1^)	Pod number (plant^−1^)	Seed weight (g `plant^−1^)	Seed number (plant^−1^)	Grain yield (t ha^−1^)	Stover yield (t ha^−1^)
SRI-156	17.50^NS^	50*	12.60^NS^	164^NS^	1.76^NS^	1.89*
SRI-158	19.33*	52*	14.43*	186*	2.01*	1.37^NS^
SRI-178	18.93*	54*	14.13*	190*	1.99*	1.44^NS^
SRI-211	18.67*	50*	13.20^NS^	177*	1.80^NS^	1.50^NS^
SRI-229	19.13*	55*	14.00*	184*	1.93*	1.91*
SRI-305	18.83*	53*	13.70*	188*	1.80^NS^	1.45^NS^
SRI-360	18.90*	47^NS^	12.63^NS^	166^NS^	1.82^NS^	1.67^NS^
Control	16.93	45	11.47	147	1.43	1.31
Mean	18.53	51	13.27	175	1.82	1.57
SE±	0.36	1.2	0.59	8.7	0.09	0.13
LSD (5%)	1.08	3.6	1.79	26.3	0.26	0.40
CV %	3	4	8	9	8	14

*SE* standard error, *CV* coefficients of variation, *NS* non-significant

* Indicates significance over the control at *p* < 0.05

**Table 7:** Effect of the seven biocontrol potential bacteria on rhizosphere soil nutrients of pigeonpea under field conditions—at harvest

Isolate	Total N (ppm)	Available P (ppm)	Organic carbon (%)
SRI-156	796^NS^	12.22*	0.56*
SRI-158	785^NS^	8.93^NS^	0.57*
SRI-178	812^NS^	7.71^NS^	0.54^NS^
SRI-211	832*	9.74^NS^	0.61*
SRI-229	866*	8.18^NS^	0.59*
SRI-305	781^NS^	9.82*	0.62*
SRI-360	870*	11.57*	0.59*
Control	774	7.58	0.52
Mean	814	9.47	0.57
SE±	15.6	0.669	0.012
LSD (5%)	52.2	2.238	0.040
CV %	3	10	3

*SE* standard error, *CV* coefficients of variation, *NS* non-significant

* Indicates significance over the control at *p* < 0.05

### Influence of selected bacterial strains on micronutrients concentration in harvested grains

When the harvested grains were evaluated for their micronutrients concentration, the plots treated with the test bacterial strains enhanced micronutrients including Fe (up to 18 and 12%), Zn (up to 23 and 5%), Cu (up to 19 and 8%), Mn (up to 2 and 39%) and Ca (up to 22 and 11%) for chickpea and pigeonpea, respectively over the un-inoculated control plots. It was noticed that, cooking of harvested grains has modified the mineral content and leads to either loss or gain of minerals on Fe (up to 19% loss; and 21% gain), Zn (up to 10% loss and 13% gain), Cu (up to 24% loss and 9% gain), Mn (up to 15% loss and 40% gain) and Ca (up to 15 and 25% gain) on both chickpea and pigeonpea (Tables 8, 9). It is understood from the table values that, raw forms holds higher density of minerals than cooked forms. Among the bacterial treatment SRI-229 in chickpea and SRI-305 in pigeonpea were found to be the best treatment in increasing the mineral density of the respective legumes by retaining the significant (*p* < 0.05) quantity of mineral in both raw and cooked forms.

**Table 8:** Effect of the seven biocontrol potential bacteria on chickpea grain mineral contents—at harvest

Isolate	Fe (mg 100 g^−1^)		Zn (mg 100 g^−1^)		Cu (mg 100 g^−1^)		Mn (mg 100 g^−1^)		Ca (mg 100 g^−1^)	
	Cook	Raw	Cook	Raw	Cook	Raw	Cook	Raw	Cook	Raw
SRI-156	4.8^NS^	5.9*	3.8*	4.2*	0.6^NS^	0.8*	3.2^NS^	3.0^NS^	132^NS^	138*
SRI-158	5.5*	5.8*	4.0*	4.1*	0.6^NS^	0.7^NS^	3.4*	3.1*	146*	155*
SRI-178	5.2*	5.7*	4.0*	4.5*	0.6^NS^	0.8*	3.4*	3.1*	132^NS^	154*
SRI-211	4.9^NS^	5.8*	4.1*	3.9*	0.7*	0.7^NS^	3.4*	3.1*	133^NS^	139*
SRI-229	5.2*	6.0*	3.9*	4.1*	0.7*	0.8*	3.4*	3.1*	137*	132*
SRI-305	5.0^NS^	5.6*	3.9*	4.0*	0.7*	0.7^NS^	3.2^NS^	3.1*	136^NS^	155*
SRI-360	5.0^NS^	6.1*	3.9*	3.9*	0.6^NS^	0.7^NS^	3.3^NS^	3.1*	134^NS^	137*
Control	4.8	5.0	3.8	3.4	0.6	0.7	3.2	3.0	131	122
Mean	5.0	5.7	3.9	4.0	0.6	0.8	3.3	3.1	136	141
SE±	0.08	0.01	0.05	0.012	0.02	0.01	0.04	0.01	1.7	2.4
LSD (5%)	0.28	0.23	0.017	0.41	0.05	0.04	0.17	0.03	5.6	8.2
CV %	2	2	2	4	3	2	2	1	2	2

*Cook* cooked harvested grains, *Raw* raw harvested grains, *SE* standard error, *CV* coefficients of variation, *NS* non-significant

* Indicates significance over the control at *p* < 0.05

**Table 9:** Effect of the seven biocontrol potential bacteria on pigeonpea grain mineral contents—at harvest

Isolate	Fe (mg 100 g^−1^)		Zn (mg 100 g^−1^)		Cu (mg 100 g^−1^)		Mn (mg 100 g^−1^)		Ca (mg 100 g^−1^)	
	Cook	Raw	Cook	Raw	Cook	Raw	Cook	Raw	Cook	Raw
SRI-156	3.6^NS^	3.5*	3.6*	4.0*	1.3*	1.2*	1.3^NS^	1.6*	116^NS^	133*
SRI-158	3.3*	3.1^NS^	3.5*	4.0*	1.2^NS^	1.2*	1.1^NS^	1.6*	117^NS^	141*
SRI-178	3.2^NS^	3.8*	3.5*	3.9*	1.3*	1.2*	1.5*	1.5*	119^NS^	125^NS^
SRI-211	3.1^NS^	3.2^NS^	3.5*	3.8*	1.3*	1.2*	1.2^NS^	1.5*	113^NS^	130*
SRI-229	3.2^NS^	3.9*	3.4^NS^	3.8*	1.3*	1.4*	1.1^NS^	1.4^NS^	115^NS^	125^NS^
SRI-305	3.4*	3.4*	3.6*	3.9*	1.3*	1.3*	1.8*	1.5*	123*	128*
SRI-360	3.3*	3.6*	3.5*	3.7^NS^	1.3*	1.2*	1.2^NS^	1.4^NS^	127*	125^NS^
Control	3.1	3.0	3.4	3.7	1.2	1.1*	1.1	1.4	113	120
Mean	3.3	3.4	3.5	3.9	1.3	1.2	1.3	1.5	118	128
SE±	0.05	0.09	0.03	0.02	0.02	0.02	0.08	0.03	2.2	2.5
LSD (5%)	0.16	0.31	0.10	0.08	0.06	0.08	0.26	0.11	7.4	8.4
CV %	2	4	1	1	2	3	9	3	3	3

*Cook* cooked grains, *Raw* raw harvested grains, *SE* standard error, *CV* coefficients of variation, *NS* non-significant

* Indicates significance over the control at *p* < 0.05

## Discussion

The bacterial strains studied (SRI-156, SRI-158, SRI-178, SRI-211, SRI-229, SRI-305 and SRI-360) in the present study were earlier demonstrated by us for biocontrol potential against *M. phaseolina*, which causes charcoal rot in sorghum, and PGP potentials in sorghum and rice (Gopalakrishnan et al. 2011, 2012). In the present study, the seven bacteria were further evaluated for their PGP and biofortification traits on grain legumes such as chickpea and pigeonpea. The influence of the selected bacterial strains on germination of both chickpea and pigeonpea was clearly demonstrated in the greenhouse where both shoot height and root length were found to be enhanced in both chickpea and pigeonpea. Among the seven bacteria, SRI-229 was found to significantly enhance both shoot height and root length in both chickpea and pigeonpea (Fig. 1). Under field conditions, the chickpea plots treated with the bacterial strains enhanced PGP traits including the nodule number, nodule weight, root weight, shoot weight, pod number, pod weight, leaf weight and leaf area and at crop maturity, yield traits including the stover yield, grain yield, pod weight, pod number and seed weight over the un-inoculated control plots. The pigeonpea plots treated with the bacterial strains also enhanced PGP traits including the nodule number, nodule weight, leaf weight, leaf area, stem weight and root weight and at crop maturity, yield traits including the pod weight, pod number, seed weight, seed number and grain and stover yields over the un-inoculated control plots. Among the tested strains, SRI-229 was found to significantly and consistently enhance all the PGP and yield traits in both chickpea and pigeonpea. The seven bacteria also enhanced rhizosphere soil nutrients including total N, available P and OC % in both chickpea and pigeonpea plots over the un-inoculated control plots. Yet again, SRI-229 was found to significantly enhance rhizospheric soil nutrients in pigeonpea but not that significant in chickpea.

In the present investigation, at 30 DAS in both chickpea and pigeonpea, the number of nodules and weight of nodules were found consistently and significantly higher in the selected bacteria-treated plots over un-inoculated control. Though, bacteria such as *Pseudomonas* sp., *Brevibacterium* sp., *Bacillus* sp., *Enterobacter* sp. and *Acinetobacter* sp. are not reported to have the nodulation capacity but its colonization on the roots and nodules and their beneficial association with native rhizobia were demonstrated to increase nitrogen fixation, nodulation, plant growth and grain yield of chickpea, soybean and pea (Tokala et al. 2002; Valverde et al. 2006; Minorsky 2008; Soe et al. 2010; Gopalakrishnan et al. 2015). The bacteria used in this study did not inhibit the growth of native rhizobia in the antagonism tests by the poisoned food technique (data not shown). Therefore, it is concluded that the selected seven bacteria are compatible with native rhizobia.

The mechanisms associated with PGP by bacteria include secretion of PGP hormones such as indole acetic acid (IAA), chelation of iron by producing compounds such as siderophore, solubilization of phosphorous and antagonistic potential against phytopathogens (Panhwar et al. 2012; Sreevidya et al. 2016). Auxins such as IAA are one of the phytohormones regulating cell differentiation, root elongation, fruit formation and abscission control (Khamna et al. 2009). Siderophores are the low molecular weight Fe-binding compounds, which binds Fe^3+^ and convert it to readily absorbable form (Gray and Smith 2005), which can be used by the plants. Phosphate solubilizing bacteria release phosphate ions from inorganic P compounds in soils and thereby contribute to an increased available phosphorous for the plants (Artursson et al. 2006). Hydrocyanic acid (HCN) is a volatile antibiotic that helps in disease suppression (Siddiqui 2006). The selected bacterial strains in this study were previously demonstrated to produce siderophore, IAA (except SRI-305), HCN (except SRI-158 and SRI-305) and solubilized (except SRI-360) phosphorous (Gopalakrishnan et al. 2011). *Bacillus* and *Pseudomonas* species employ an array of mechanisms including antibiosis, HCN and siderophore production, antifungal metabolites, fluorescent pigments and competition to antagonize pathogens (Validov et al. 2005; Singh et al. 2006). Hence it is concluded that one of these mechanisms could be the reason for their PGP traits.

Bacteria having broad spectrum PGP potentials are reported widely in literature. Bacteria belonging to genera *Bacillus*, *Serratia*, *Enterobacter* and *Pseudomonas* are widely reported to solubilize the insoluble phosphorous and help in plant growth (Rodriguez and Fraga 1999). PGP bacteria such as *Bacillus subtilis* BN1 from the rhizospheres of chir pine (*Pinus roxburghii*), fluorescent *Pseudomonas* GRC_2_ from potato rhizosphere and *Pseudomonas chlororaphis* SRB 127 from sorghum rhizosphere showed strong antagonistic effect against *M. phaseolina*, a charcoal rot pathogen of sorghum and peanut (Gupta et al. 2002a, b; Das et al. 2008; Singh et al. 2008). Enhanced plant growth and soil nutrient traits has been reported on various species of *Pseudomonas* and/or *Bacillus* on grain legumes such as mung bean (Gupta et al. 2002a, b; Tripathi et al. 2005), soybean (Gupta et al. 2005), black gram (Ganesan 2008) and chickpea (Tank and Saraf 2009; Wani and Khan 2010; Gopalakrishnan et al. 2015).

The selected seven bacteria were also able to grow at pH levels between 5 and 13, temperatures between 20 and 40 °C and salinity (NaCl) up to 6% under in vitro conditions and thus have the ability to survive under harsh environments such as saline and acidic to alkaline soils (Gopalakrishnan et al. 2012). The interaction between native or introduced soil bacteria and roots and their possible impacts on plant growth have been extensively reported by Birkhofer et al. (2008) and Uphoff et al. (2009). It can be concluded that the bacterial isolates studied in this investigation were apparently well adapted to the field conditions of chickpea and pigeonpea, in addition to their adaptability in sorghum and rice rhizosphere environments (from our previous studies).

In the present study, when the harvested grains as well as processed grains (in order to know mineral availability of edible forms) of both chickpea and pigeonpea were evaluated for their micronutrients concentration, the plots treated with the test bacterial strains enhanced micronutrients of both type (harvested and processed) on Fe, Zn, Cu, Mn and Ca over the un-inoculated control plots. This increase might be due to their mineral mobilizing ability through the production of siderophores which was reported in our earlier studies (Gopalakrishnan et al. 2011). Siderophores play as solubilizing agents for iron under iron limitation conditions (Indiragandhi et al. 2008). Further, siderophores forms stable complexes with heavy metals such as Ga, Zn, Al, Cu, U, Np, Cd, Pb and In and helps to alleviate the metal stresses imposed on plants (Rajkumar et al. 2010). Rana et al. (2012a, b) observed that a combination of PGP *Bacillus* sp. AW1 and *Providencia* sp. AW5 increased the mineral of content up to 105% with higher counts for Fe, Zn and P on harvested rice and wheat grains.

The other possible reason for increased mineral contents could be, modification of root system (increased root length, weight and volume) by the same set of test bacteria as observed on our previous studies on rice (Gopalakrishnan et al. 2012). Sessitsch et al. (2013) also approved this hypothesis by demonstrating the influence of enhanced root system on uptake of trace elements. In the present study, similar observations could not be collected as under field conditions, the roots of chickpea starts degrading at the time of harvesting and is difficult to collect the roots. Besides this, other mechanisms, including organic acids, polymeric substances, biosurfactants, and oxidation reduction reactions might also influence the mineral availability in the root-soil interface and hence increased mineral availability (Ma et al. 2011).

## Conclusion

The use of PGP bacteria has increased in many parts of the world due to their significant contribution in growth and yield in crops such as wheat, rice, tomato, bean, pea and chickpea (Tokala et al. 2002; Nassar et al. 2003, El-Tarabily 2008, Sadeghi et al. 2012 Gopalakrishnan et al. 2015). PGP bacteria also are reported to have induced systemic resistance against a broad range of plant pathogens and insect pests (Jetiyanon and Kloepper 2002; Ryu et al. 2007). The present study has demonstrated the PGP potentials of the selected bacteria in chickpea and pigeonpea under field conditions and thus can be a useful component of integrated plant health and disease management. This study further suggests the use of PGP bacterial inoculums could end in development of a complementary sustainable tool for the influence of existing biofortification strategies. Of the seven bacteria studied in the current investigation, SRI-229 was found superior to other bacteria in terms of their effects on root and shoot development, nodule formation, crop productivity and soil nutritional factors followed by SRI-158 and SRI-305. The usage of such broad spectrum PGP bacteria with multiple actions for crop production and protection is novel as with one biological treatment/application controls more than one problems apart from promotion of plant growth. Further studies are required to determine the actual mechanisms behind the mineral transfer from soil to seed and the effectiveness of these bacteria under different field conditions by conducting multi-location trials. Development of such beneficial microbial inoculum as bio-fertilizer can serve as an agro-input in organic farming systems of various crops.
